# Adherence to diabetic retinopathy screening among children and young adults in Bangladesh

**DOI:** 10.1186/s40842-024-00208-2

**Published:** 2024-12-04

**Authors:** Katie Curran, Munir Ahmed, Mirza Manbira Sultana, Salissou Moutari, Mohammad Awlad Hossain, Laura Cushley, Tunde Peto, Lutful Husain, Bedowra Zabeen, Nathan Congdon

**Affiliations:** 1 Faculty of Medicine Health and Life Sciences, Queen’s University Belfast, Great Britain and Northern Ireland, Belfast, UK; 2 Orbis International, Dhaka, Bangladesh; 3 School of Mathematics and Physics, Queen’s University Belfast, Britain and Northern Ireland, Belfast, UK; 4 Orbis International, New York, USA; 5 BADAS Paediatric Diabetes Care and Research Centre, Dhaka, Bangladesh; 6 Institute of Research and Rehabilitation in Diabetes, Endocrine and Metabolic Disorders (BIRDEM), Diabetic Association of Bangladesh (BADAS), Dhaka, Bangladesh; 7 Zhongshan Ophthalmic Centre, Sun Yat-sen University, Guangzhou, China

**Keywords:** Diabetes mellitus, Diabetic retinopathy, Children, Young adults, Screening

## Abstract

**Background:**

Effective diabetic retinopathy screening (DRS) programmes are important in preventing vision impairment and blindness caused by diabetes. This study focuses on identifying the factors affecting attendance or non-adherence to DRS among children and young adults with diabetes mellitus (DM) in Bangladesh.

**Methods:**

A mixed-methods approach was used, which included patients diagnosed with DM aged 12–26 years from Bangladesh who were registered at BIRDEM Women and Children hospital in Dhaka. Data collection occurred between July 2019 and July 2020, mainly through telephone and email due to restrictions imposed by the COVID-19 pandemic. Statistical analyses, including chi-squared tests, t-tests, and logistic regression, were used to assess the demographic and clinical factors influencing attendance at DRS.

**Results:**

The study reported a high 88% attendance rate for DRS among children and young adults in Bangladesh. However, some barriers to attendance were identified. Children under 15 years of age showed a higher tendency to attend their last DRS appointment when compared to older age participants (16–26 years), *P* < 0.05. Male participants demonstrated a lower likelihood of attending their DRS appointments than females (OR 0.29, CI: 0.17 to 0.50), *P* < 0.001. Additionally, participants with higher HbA1c levels (mean 9.1%, IQR 2.5) attended their last DRS appointment compared to those with lower levels (mean 8.0%) (*p* < 0.05). The primary barriers leading to missed DRS appointments were distance to the hospital (15, 31.9%), financial limitations (19, 40.4%), and busy schedules (14, 29.8%).

**Conclusions:**

Compliance with DRS was high in this setting especially among younger patients, females, and those with higher HbA1c levels, highlighting the effectiveness of current DRS initiatives in Bangladesh. Addressing barriers such as cost, service accessibility and transportation could improve attendance rates further, and strategies such as flexible scheduling, transport subsidies, telemedicine, and use of artificial intelligence may help overcome these challenges.

## Background

Type 1 diabetes mellitus (T1DM) is on a continual upward trend each year. Globally, among 2.61 billion children and young people (< 20 years), 1.21 million are affected by T1DM, a prevalence rate of approximately 0.05% [1]. In Bangladesh, the prevalence of T1DM among those under 20 years is estimated at 0.01%, with 5,719 cases reported [1]. Concurrently, there is a global rise in type 2 DM (T2DM), partly attributable to widespread increases in childhood obesity [2]. Populations with high prevalence of T2DM in children and young people tend also to have a higher risk of adult T2DM [2]. The increase in T2DM prevalence in young adults is particularly pronounced in South Asian ethnic groups [3–5].

Diabetic retinopathy (DR) is a common microvascular complication of DM, posing a significant threat to vision, particularly in young people with early onset, whose lifetime risk is increased [6]. Early stages of DR are often asymptomatic, making regular screening necessary for early detection and intervention [7]. This is especially important in younger cohorts, where early onset and potentially rapid progression of DM complications can occur. The prevalence of DR in children and young adults varies significantly across regions, and is influenced by factors such as the duration of DM, blood sugar control, and access to healthcare services [2, 8]. A 2023 study revealed that the global prevalence of DR among children and adolescents with T2DM was 6.99%, with DR increasing significantly with DM duration in excess of 5 years [9].

The prevention of vision impairment and blindness in children and young adults with DM requires a comprehensive and multidisciplinary approach, including counselling, dietary guidance, blood pressure monitoring, and tracking body mass index (BMI). Stressing the significance of effective DM management is crucial for reducing the risk of both the onset and progression of DR. Moreover, regular diabetic retinopathy screening (DRS) is recommended, with the specific guidelines depending on the local context and healthcare system in place.

Transitioning young patients with DM from paediatric to adult care is a pivotal phase in their healthcare journey. This transition must be carefully coordinated to ensure that their medical, educational, psychosocial, and occupational needs are adequately met [10, 11]. Preparing adolescents early to take responsibility for their own care is essential for long-term management. Without a well-planned transition, there can be adverse consequences, including an increased risk of hospitalisation when transitioning between healthcare services and providers [10, 11].

In Bangladesh, the Diabetic Association of Bangladesh (BADAS) plays a significant role in providing comprehensive care for children and young adults with diabetes through its Pediatric Diabetes Care and Research Centre (PDRC), with support from programmes “Life for a Child” (LFAC) and “Changing Diabetes in Children” (CDIC) [12]. BADAS -PDRC has made substantial progress in increasing awareness about DM and ensuring access to treatments, even for the poorest individuals. BADAS operates on a cross-financing model, where those who are financially privileged subsidise care for the economically disadvantaged [12]. However, the rising prevalence of DM in Bangladesh has put a strain on BADAS’s resources. In response, the collaboration with the LFAC programme was aimed at providing support, including regular check-ups and DRS.

Orbis International has partnered with BADAS to introduce a scalable model for DRS into existing DM care models for children and young adults. The aim is to reduce the burden of blindness and vision impairment among children and young adults in low-resource settings, marking it as a pioneering effort on a global scale [13].

Attendance at DRS is influenced by various elements such as socio-economic status, DM awareness, and access to healthcare [14, 15]. In high-income areas, structured screening programmes and public health initiatives have been shown to enhance DRS attendance. However, the scenario in low-middle income countries (LMICs) is more complicated, due to factors such as financial barriers, which can hinder access to DRS owing to costs related to screening, transportation, and treatment [15]. Geographical constraints, particularly in remote or rural areas, limit access to healthcare services, affecting regular DRS attendance. Additionally, cultural norms, stigmas, social influences, and misconceptions about eye health may prevent attendance at DRS [15]. In some LMICs, women and girls encounter difficulties in accessing eye care due to social responsibilities, which can also limit their educational and employment opportunities [16]. In Bangladesh, a comprehensive holistic DM clinic is available for children and young adults, with a Paediatric Endocrinologist, Nutritionist, Psychologist, Ophthalmologist and DM educator all in the one location. This setup may enhance attendance due to parental involvement and eliminates the need for multiple visits to different clinics.

Effective DRS models are crucial for preventing DR-related vision impairment and blindness in children and young adults, and they require optimal attendance to be effective [12]. There is a need to understand compliance rates with such programmes among young people in Bangladesh and other LMICs to identify factors that enable and hinder their attendance. The lack of research focusing on factors influencing DRS attendance among the young in LMICs highlights the need for more studies in this area.

## Objectives

The primary objective of this study was to investigate the factors associated with adherence and non-adherence to DRS among children and young people with DM in Bangladesh.

### Methods

#### Study design

This research used a mixed-methods approach, incorporating a structured questionnaire with predominantly closed-ended questions in Bengali. A few open-ended options were also included to allow participants to respond in their own words. Before the full data collection, a pilot questionnaire was administered to a smaller group of 30 participants to identify any issues with the questions or response format. Based on the findings from the pilot study, any necessary changes or amendments to the questionnaire were made.

Participants: BIRDEM Women and Children Hospital in Dhaka, Bangladesh, is a multi-specialty hospital specialising in maternal and child health. The BADAS- PDRC centre within the hospital provides comprehensive care for children and young people with DM (aged 1–26 years). Orbis, a non-governmental organisation dedicated to eye care, has integrated a DRS component into the PDRC centre. Study participants were enrolled as patients coming to the PDRC centre, according to the following criteria:

Inclusion criteria:

- Patients diagnosed with DM aged 12–26 years who visited PDRC centre for an appointment.
- Caregivers willing to participate in the study.

### Data collection

#### Pilot study

The pilot phase was conducted from July-September 2019 to address and overcome challenges encountered during data collection. One notable adjustment involved the inclusion of a marital status question, considering that approximately 69% of young women are married before the age of 18 years in Bangladesh [17]. We also included supplementary questions on the impact of COVID-19 on adherence to DRS. These refinements in data collection parameters contributed to a more comprehensive understanding of the factors influencing DRS adherence.

Data collection for the main study was conducted by telephone and email between June and July 2020. Since travel was restricted due to the COVID-19 pandemic, face-to-face interviews were not possible. Participants who missed their last appointment were contacted and asked the same questions as those who attended, using a standardised questionnaire. A systematic random sampling method (determined by dividing the total population by required sample size) was applied to select the study participants from a list of patients registered with PDRC, at BIRDEM. In cases, we where we were unable to reach potential participants, we selected the next participants on the list.

#### Statistical analysis

All data were entered into Microsoft Excel (version 2021, Microsoft Corporation, Redmond, Washington). Participants were categorised into attendee’s vs. non-attendees based on their last DRS appointment. Demographic and clinical parameters of participants in each group were analysed. Categorical and continuous variables were presented as frequencies (percentages), mean (standard deviation [SD]) or noncontinuous variables as median (interquartile range [IQR]). Chi square test was used to compare the distributions of the categorical variables (with two levels or more) between the patients who attended last DRS appointment and those who didn’t, whereas the t-test was used to compare two proportions and continuous variables. Univariate and multivariate ordinal logistic regression analysis was performed to investigate the effects of the potential predictors on DRS attendance. Variables which were statistically significant (*p* ≤ 0.05) in the univariate model were included in the final multivariate logistic regression model. The results of attendance to the last DRS appointment are highly imbalanced with 88% (*n* = 343) of attendance and 22% (*n* = 47) of non-attendance. To build a robust logistic regression model, these data need to be balanced. Balancing imbalanced data is a critical step in preparing datasets for statistical modelling, especially when dealing with classification problems where one class significantly outnumbers the others. Imbalanced data can lead to biased models that perform poorly on the minority class. Data-level techniques are generally used to address this issue by modifying the training data to balance the class distribution. The most commonly used data-level techniques include; (i) *oversampling*, which consists of increases the number of instances in the minority class by randomly duplicating them or generating new synthetic examples; (ii) *undersampling*, which consists of random reduction of the number of instances in the majority class to balance the class distribution; (iii) *hybrid sampling*, which combines both oversampling and undersampling to balance the dataset while mitigating the risk of overfitting due to oversampling or loss of information due to undersampling. In this study, we use the hybrid sample method to generate the balanced training set, which is used to build the logistic regression models.

All analyses were conducted using Python Software Foundation (Python Language Reference, version 3.9, Available at http://www.python.org).

## Results

Responses were analysed for 390 participants. Demographic characteristics and clinical parameters are presented separately for participants who did and did not attend their last DRS appointment (Table 1). Only 47/390 (12%) participants missed their last DRS appointment. Younger participants (mean age 17.7 years) and females (51.6%) were more likely to have attended their last DRS appointment, with most attendees coming from Dhaka (68.5%). Those who missed their last DRS appointment were slightly older (mean age 19 years, IQR:6) and a higher proportion were males (70.2%) (*P* < 0.05). The majority of participants in both groups were Muslim (94.2% of attendees,97.9% of non-attendees).

**Table 1:** Characteristics of children/young adults with diabetes in Bangladesh, stratified by Diabetic Retinopathy Screening attendance

Characteristics of young person	Attended last DRS appointment (*n* = 343)	Did not attend last DRS appointment (*n* = 47)	*p*-value
Socio-demographic Parameters			
Age, n(%) - Under 15 - 16–19 - 20–22 - 23–26 Age, n(%) - Mean (SD)	110(32.1%) 123(35.9%) 64(18.7%) 46(13.4%) 17.7 (3.8)	10(21.3%) 14(29.8%) 14(29.8%) 9(19.1%) 19.0 (3.9)	0.137 0.030*
Gender, n (%) Female Male	177(51.6%) 166(48.4%)	14 (29.8%) 33 (70.2%)	0.008*
Religion, n (%) - Muslim - Christian - Hindu	323(94.2%) 1(0.3%) 19(5.5%)	46(97.9%) 0(0.0%) 1(2.1%)	0.567
Mode of transport to hospital, n (%) (multiple response) - Rickshaw, - three-wheeler/Auto - Van - Private Car/Rented Car - Boat - Ship - Bus - Train - Other (walking)	128(37.3%) 111(32.4%) 7(2.0%) 14(4.1%) 4(1.2%) 15(4.4%) 245(71.4%) 31(9.0%) 121(35.3%)	15(31.9%) 13(27.7%) 0(0%) 2(4.3%) 0(0.0%) 0(0.0%) 33(70.2%) 4(8.5%) 15(31.9%)	0.894
Distance to hospital (time in minutes) (Median ± IQR)	130 ± 185	120 ± 310	0.852
Distance to hospital (km) (Median ± IQR)	35 ± 112	40 ± 120	0.813
Total family household members (Median ± IQR)	5 ± 1	5 ± 2	0.181
Education Level Status, n (%) - Illiterate/no school attendance - Primary Education Certificate - Junior School Certificate - Secondary School Certificate - Higher Secondary School Certificate and above	8(2.3%) 67(19.5%) 103(30.0%) 80(23.3%) 85(24.8%)	4(8.5%) 10(21.2%) 12(25.5%) 9(19.1%) 12(25.5%)	0.215
Occupation of young person, n (%) - Day Labour/Farmer - Service (Government and Private) - Student - Small business/business - Housemaker - Unemployment - Others	10(2.9%) 11 (3.2%) 275(80.2) 5(1.5%) 22 (6.4%) 16(4.7%) 4(1.2%)	5(10.7%) 2(4.3%) 32(68.1%) 1(2.1%) 1(2.1%) 2(8.5%) 2(4.3%)	0.071
Household Heads Occupation, n (%) - Day Labour/Farmer - Service (Government and Private) - Driver/Rickshaw puller - Small business/business - Housemaker - Unemployment - Others	82(23.9%) 121(36.3%) 10(2.9%) 94(27.4%) 19(5.5%) 10(2.9%) 7 (2.0%)	11(23.4%) 17(36.1%) 4(8.6%) 13(27.6%) 0(0.0) 1(2.1%) 1(2.1%)	0.389
Currently married, n (%)	34(9.9%)	4(8.5%)	0.761
Family monthly income (US Dollar) (Median ± IQR)	180 ± 180	135 ± 180	0.265
Family receives financial Government support, n (%)	22(6.4%)	1(2.1%)	0.242
Clinical Parameters and DM Management			
Age of DM diagnosis (years), n (%) - 5 or Under - 6–10 - 11–16 - 17 or Over (Median ± IQR)	28(8.2%) 103(30.0%) 193(56.3%) 19(5.5%) 12 ± 4	4(8.5%) 16(34.0%) 22(46.8%) 5(10.6%) 12 ± 5	0.450 0.777
DM Type, n (%) - Type 1 - Type 2 - Unknown - Nonresponse	155(45.2%) 53(15.5%) 134(39.1%) 1(0.3%)	23(48.9%) 4(8.5%) 20(42.6%) 0(0.0%)	0.625
DM controlled by; n (%) (multiple response) - Insulin - Other hypoglycaemic medication - Insulin and hypoglycaemic medication - Diet/Exercise - Other	298 (86.9%) 63(18.4%) 0(0.0%) 291(84.8%) 261(76.7%) 0(0.0%) 0(0.0%)	45(95.7%) 9(19.1%) 0(0.0%) 41(87.2%) 39(82.9%) 2(4.3%) 0(0.0%)	0.009*
DM duration (years), (Median ± IQR)	5 ± 5	7 ± 5	0.212
HbA1c at last test (Median ± IQR)	8.0 ± 2.5	9.1 ± 2.5	0.010*
How often are blood sugars tested at home, n (%) - Daily - 1-3x a week - 4-6x a week - Others - Nonresponse	41(12.0%) 221(64.5%) 31(9.0%) 26(7.6%) 24(7.0%)	11(23.4%) 21(44%) 4(8.5%) 4(8.5%) 7(14.9%)	0.041*
First degree relatives with DM (mother, father, brother, sister), Y, n (%)	187(54.5%)	17(36.2%)	0.018*
Any DM complications, Y, n (%) (multiple response) - Foot trouble, - Neuropathy - Eye problems - Heart problems - Stroke - Teeth problem - Very high blood sugars (hyperglycaemia) - Very low blood sugars (hypoglycaemia) - Skin problems - Not sure - Others	61(17.8%%) 7(2.0%) 136(39.7%) 7(2.0%) 1(0.3%) 55(16.0%) 25(7.3%) 172(50.1%) 23(6.7%) 22(6.4%) 68(19.8%)	8(17.0%) 2(4.3%) 14(29.8%) 4(8.5%) 0(0.0%) 3(6.4%) 3(6.4%) 26(55.3%) 1(2.1%) 4(8.5%) 18(38.3%)	0.898 0.343 0.192 0.012* 0.711 0.081 0.822 0.506 0.221 0.589 0.004*
Last attendance at hospital for DM treatment, n (%) - 1 week ago - 2 weeks ago - 3 weeks ago - 1 month ago - 3 months ago - 6 months ago - Other	4(1.2%) 4(1.2%) 0(0.0%) 44(12.8%) 240(73.3%) 31(9.0%) 20(5.8%)	2(4.3%) 0(0.0) 0(0.0%) 8(17.0%) 17(36.2%) 4(8.5%) 16(34.0%)	< 0.001*
Attended any hospital follow-up visits in the last 6 months, Y, n (%)	207(60.3%)	23(48.9%)	0.136

Missing: *n* = 12

*Statistically significant at 5% level (p-value < 0.05)

The primary mode of transport to the hospital for both groups was by bus (> 70%), however, no statistically significant differences were observed. Attendees, on average, took slightly longer to reach their appointments (130 min) compared to non-attendees (120 min) (*P* > 0.05). In terms of educational differences, only 2.3% of attendees were reported as illiterate compared to 8.5% of non-attendees (*P* < 0.05). Attendees reported a slightly higher average monthly family income of 20,000 Taka (equivalent to £143.94) compared to the 15,000 Taka (approximately £107.95) reported by non-attendees (*P* > 0.05). Non-attendees displayed a longer mean duration of DM (8 years, IQR: 5) in contrast to attendees (7 years, IQR: 5) (*P* > 0.05), and non-attendees had a higher mean HbA1c result at their last test (9.1, SD: 2.5) compared to attendees (8.0, SD: 2.5) (*P* < 0.05).

Reasons for missing previous DRS appointments in Bangladesh included financial constraints, distance to the hospital, and busy schedules, with these factors being more prominent among the defined non-attendee group. Overall, the majority of participants rated hospital treatment and services as either very good or good (Table 2).

**Table 2:** Survey responses on diabetic eye care for children/young adults in Bangladesh

Diabetic Eye Care Survey Responses	Attended last DRS appointment. (n = 343)	Did not attend last DRS appointment. (n = 47)	P-value
Eye problems present, n (%)	164(47.8%)	23(48.9%)	0.885
Do you think your eyes are damaged by DM? (n, %) - Yes - No - Not sure	206(60.1%) 96(28.0%) 41(12.9%)	24(51.1%) 17(36.2%) 6(12.8%)	0.462
Advised to attend follow up visit for diabetic retinopathy screening, Y, (n, %)	342(99.7%)	38(80.9%)	< 0.001*
If you missed any DRS appointments, why was this? n (%) (multiple response) - Distance to hospital - Financial Constraints - Lack of transportation - Fear of Contracting Diseases - Forgot to attend - Lack of Accompanying Person - Exam or class schedule - Mental Health Issues - Physical Health Issues - Busy schedule - Family problem - Doctors negative attitude - Poor service - Others	11(3.2%) 15(4.4%) 0(0.0%) 1(0.3%) 0(0%) 4(1.2%) 3(0.9%) 2(0.6%) 0(0%) 9(2.6%) 0(0.0%) 2(0.6%) 1(0.3%) 5(1.5%)	15(31.9%) 19(40.4%) 2(4.3%) 3(6.4%) 0(0.0%) 2(4.3%) 5(10.6%) 0(0.0%) 3(6.4%) 14(29.8%) 2(4.3%) 3(6.4%) 2(4.3%) 14(2.8%)	< 0.001* < 0.001* < 0.001* < 0.001* -- 0.107 < 0.001* 0.600 < 0.001* < 0.001* < 0.001* 0.001* 0.004* < 0.001*
How would you rate hospital treatment and services? n (%) - Very good - Good - Average - Poor - Very poor	166(48.4%) 168(49.0%) 7(2.0%) 2(0.6%) 0(0.0%)	24(51.1%) 21(44.7%) 2(4.3%) 0(0.0%) 0(0.0%)	0.712

Abbreviations: Y: Yes, DM: DM mellitus

*Statistically significant at 5% level (p-value < 0.05)

A higher proportion of attendees strongly agreed (27.4%) on the hereditary nature of DM and the association of poor diet and insufficient exercise as risk factors for DM, compared to non-attendees (6.4%) (*P* < 0.005) (Table 3). Notably, a similar percentage of participants in both cohorts either strongly or moderately agreed that individuals with DM are more likely to develop eye complications compared to those without (*P* > 0.05) (Table 3).

**Table 3:** Children and young adults’ knowledge of diabetes and diabetic retinopathy in Bangladesh

Knowledge of diabetes and diabetic retinopathy	Attended last DRS appointment (n = 343)	Did not attend last DRS appointment (n = 47)	p-value
DM is hereditary: - Strongly agree - Moderately agree - Undecided - Moderately disagree - Strongly disagree	94(27.4%) 55(16.0%) 79(23.0%) 38(11.1%) 77(22.4%)	3(6.4%) 7(14.9%) 7(14.9%) 22(46.8%) 8(17.0%)	< 0.001*
Poor diet and lack of exercise is a risk factor for DM: - Strongly agree - Moderately agree - Undecided - Moderately disagree - Strongly disagree	180(52.5%) 79(23.0%) 31(9.0%) 26(7.6%) 27(7.9%)	13(27.7%) 22(46.8%) 7(14.9%) 4(8.5%) 1(2.1%)	0.001*
People with DM are more likely to develop eye complications compared to those without: - Strongly agree - Moderately agree - Undecided - Moderately disagree - Strongly disagree	186(54.2%) 83(24.2%) 48(14.0%) 130(3.8%) 13(3.8%)	21(44.7%) 15(31.9%) 4(8.5%) 4(8.5%) 3(6.4%)	0.248

Abbreviations: DM: DM mellitus

*Statistically significant at 5% level (p-value < 0.05)

Participants were also surveyed about their DM management during the COVID-19 pandemic in Bangladesh. Only 44.6% accessed treatment services during this period, with visiting nearby medicine shops or pharmacies being the most common method of care (24.8%). Challenges faced during the pandemic included missing follow-up visits due to travel constraints for treatment (Table 4).

**Table 4:** Diabetes and diabetic retinopathy management for children/young adults during the COVID-19 pandemic in Bangladesh

DM and diabetic retinopathy management during the COVID-19 Pandemic	Attended last DRS appointment (n = 343)	Did not attend last DRS appointment (n = 47)	p-value
Accessed DM/DR treatment services during the COVID-19 pandemic, Y, n (%)	153(44.6%)	10(21.3%)	0.002*
What treatment services did you access during the COVID-19 pandemic, n (%) - Telemedicine service - BADAS Centres - BIRDEM Women and Children Hospital - Visited nearby Medicine shop/Pharmacy, - Government hospitals (UHC/Sadar Hospital) - MBBS doctor - Private Hospital - Eye Hospital - NGO hospital (non-profit) - Guidance of BIRDEM - Courier from PDRC - Others	35(10.2%) 14(4.1%) 25(7.3%) 85(24.8%) 4(1.0%) 7(2.0%) 16(4.7%) 3(0.9%) 0(0.0%) 19(5.5%) 0(0.0%) 1(0.3%)	3(6.4%) 0(0.0%) 2(4.3%) 4(8.5%) 0(0.0%) 0(0.0%) 1(2.1%) 0(0.0%) 0(0.0%) 1(2.1%) 0(0.0%) 1(2.1%)	0.337
Difficulties with DM treatment during COVID-19 pandemic, Y, n (%)	230(67.1%)	19(40.4%)	< 0.001*
What type of difficulties did you face when managing your DM during COVID-19 pandemic, n (%) (multiple response) - Missing follow up visit - No access to insulin - Struggled to monitor blood sugar levels consistently - Poor diet control - Poor exercise control - Missed taking regular oral medication - Couldn’t travel for treatment - Routine services from BIRDEM were not available - Others	180(52.5%) 88(25.7%) 12(3.5%) 34(9.9%) 69(20.1%) 3(0.9%) 168(49.0%) 0(0.0%) 1(0.3%)	13(27.7%) 13(27.7%) 3(6.4%) 1(2.1%) 1(2.1%) 0(0.0%) 14(29.8%) 0(0.0%) 0(0.0%)	0.769 0.335 0.080 0.003* 0.520 0.013* -- 0.711 0.075

Abbreviations: Y: Yes, DM: DM mellitus, DR: diabetic retinopathy,

BADAS: Diabetic Association of Bangladesh, NGO: Non-Governmental Organisation,

MBBS: Bachelor of Medicine-Bachelor of Surgery, CDiC: Changing DM in Children Programme

*Statistically significant at 5% level (p-value < 0.05)

The logistic regression analysis revealed key determinants affecting attendance to DRS, as detailed in Table 5. Children under 15 years of age were significantly more likely to keep their last DRS appointment when compared to older age participants (16 to 26 years) (*P* < 0.005). In contrast, male participants were less likely to attend DRS than females, as indicated by both univariate (OR 0.39, CI: 0.28 to 0.53) and multivariate analyses (OR 0.29, CI: 0.17 to 0.50), *P* < 0.001.

Our findings also showed that individuals with T2DM were nearly three times more likely to attend their DRS appointments than those with T1DM. Similarly, participants who had been living with DM for a shorter duration were more likely to attend their DRS appointments than those diagnosed for more than five years (OR 0.53, CI: 0.28 to 0.98). Although distance to the hospital was commonly cited as a barrier to accessing DRS, no statistically significant differences were observed among the groups. Notably, individuals residing more than 30 min from the DRS centre, especially those living 30–60 km and beyond 120 km, were more likely to attend their appointments (*P* < 0.005).

Educational level significantly influenced attendance; individuals with a Secondary Certificate were 11 times more likely to attend DRS (OR 11.01, CI: 2.36 to 51.28), and those with a Higher Secondary School Certificate or higher were 30 times more likely (OR 30.34, CI: 5.96 to 155.48), despite the wide confidence intervals. Income also played a role, with those earning 10–25,000 taka showing lower attendance rates compared to the under 10,000-taka group (OR 0.44, CI: 0.21 to 0.96). While those earning over 25,000 takas had a higher attendance rate than the lowest income group, this difference was not statistically significant. Lastly, having family financial support significantly increased the likelihood of attending DRS, with more than a sixfold increase (OR: 6.36, CI: 1.64 to 24.67) Table 5.

**Table 5:** Logistic regression - univariate and multivariate analysis

	Univariate Analysis		Multivariate Analysis	
Participant Characteristics	p-value	OR (95% CI)	p-value	OR (95% CI)
**Age (years)**				
16–19 (vs. 15 or less)	0.083	0.70 (0.47; 1.05)	0.003*	0.38 (0.21; 0.72)
20–22 (vs. 15 or less)	< 0.001*	0.42 (0.27; 0.66)	< 0.001*	0.08 (0.03; 0.20)
23–26 (vs. 15 or less)	< 0.001*	0.35 (0.22; 0.57)	< 0.001*	0.09 (0.03; 0.28)
**Gender Male (vs. Female)**	< 0.001*	0.39 (0.28; 0.53)	< 0.001*	0.29 (0.17; 0.50)
**DM type**				
Type 2 (vs. Type 1)	< 0.001*	2.68 (1.55; 4.60)	0.009*	3.09 (1.32; 7.23)
Unknown (vs. Type 1)	0.841	1.03 (0.75; 1.42)	0.356	1.26 (0.77; 2.08)
**Diabetes duration (years)**				
6–10 (vs. 5 or less)	< 0.001*	0.49 (0.36; 0.66)	0.044*	0.53 (0.28; 0.98)
11 or more (vs. 5 or less)	0.489	1.34 (0.58; 3.10)	0.771	1.23 (0.30; 4.97)
**Religion**				
Christian (vs. Islam)	--	--	--	--
Hindu (vs. Islam)	0.022*	2.79 (1.16; 6.73)	0.009*	7.71 (1.65; 35.98)
**Residence**				
Barisal (vs. Dhaka)	0.007*	0.21 (0.07; 0.65)	< 0.001*	0.01 (0.001; 0.05)
Chittagong (vs. Dhaka)	0.004*	0.51 (0.32; 0.81)	0.009*	0.35 (0.16; 0.77)
Khulna (vs. Dhaka)	0.331	1.51 (0.66; 3.46)	0.096	2.87 (0.83; 9.93)
Rajshahi (vs. Dhaka)	0.013*	3.52 (1.31; 9.46)	0.002*	8.8 (2.24; 34.59)
Rangpur (vs. Dhaka)	0.001*	0.42 (0.25; 0.71)	0.486	0.72 (0.29; 1.81)
Sylhet (vs. Dhaka)	0.002*	0.14 (0.04; 0.49)	< 0.001*	0.03 (0.01; 0.18)
**Distance to hospital (km)**				
20–50 (vs. 20 or less)	0.194	0.75 (0.48; 1.16)	0.866	1.08 (0.46; 2.54)
50–100 (vs. 20 or less)	0.942	0.98 (0.57; 1.68)	0.759	1.21 (0.36; 3.99)
More than 100 (vs. 20 or less)	0.090	0.74 (0.52; 1.05)	0.601	0.73 (0.23; 2.34)
**Distance to hospital (time in minutes)**				
30–60 (vs. 30 or less)	0.054	1.68 (0.99; 2.85)	< 0.001*	6.96 (2.83; 17.12)
60–120 (vs. 30 or less)	0.405	1.27 (0.73; 2.22)	0.088	2.37 (0.88; 6.40)
More than 120 (vs. 30 or less)	0.285	1.29 (0.81; 2.03)	0.002*	6.67 (1.96; 22.67)
**Education level**				
Illiterate/no school attendance (Reference)				
Junior School Certificate	< 0.001*	5.37 (2.34; 12.31)	0.129	3.17 (0.71; 14.03)
Primary Education Certificate	0.002*	3.67 (1.58; 8.53)	0.103	3.36 (0.78; 14.46)
Secondary School Certificate	< 0.001*	4.65 (2.01; 10.76)	0.002*	11.01 (2.36; 51.28)
Higher Secondary School Certificate and above	0.001*	3.86 (1.68; 8.86)	< 0.001*	30.34 (5.92; 155.48)
**Occupation of child/young adult**				
Unemployment (Reference)				
Day Labour/Farmer	0.482	0.71 (0.28; 1.83)	0.093	0.26 (0.05; 1.26)
Housemaker	< 0.001*	11.0 (3.24; 37.36)	0.023*	6.72 (1.31; 34.57)
Service (Government and Private)	0.445	1.47 (0.55; 3.92)	0.057	0.27 (0.07; 1.04)
Small business/business	0.578	0.71 (0.22; 2.33)	0.42	0.49 (0.09; 2.79)
Student	0.005*	2.43 (1.30; 4.54)	0.005*	0.19 (0.06; 0.60)
Others	0.169	0.42 (0.12; 1.45)	0.003*	0.05 (0.01; 0.37)
**Occupation of household head**				
Unemployment (Reference)				
Day Labour/Farmer	0.461	0.68 (0.25; 1.88)	0.517	0.57 (0.11; 3.11)
Driver	0.257	0.47 (0.13; 1.74)	< 0.001*	0.02 (0.001; 0.18)
Driver/Rickshaw puller	0.024*	0.13 (0.02; 0.76)	0.012*	0.03 (0.001; 0.46)
Service (Government and Private)	0.484	0.70 (0.26; 1.90)	0.005*	0.09 (0.02; 0.47)
Small business/business	0.199	0.51 (0.19; 1.42)	0.044*	0.18 (0.03; 0.95)
Large business	0.029*	4.9 (1.18; 20.37)	0.608	0.59 (0.08; 4.52)
Housemaker	--	--	--	--
Others	0.306	0.49 (0.13; 1.92)	0.007*	0.06 (0.01; 0.46)
**Family income (in 1000 of Taka)**				
10–25 (vs. Less than 10)	< 0.001*	0.39 (0.24; 0.65)	0.038*	0.44 (0.21; 0.96)
25–50 (vs. Less than 10)	0.369	0.77 (0.44; 1.36)	0.352	1.55 (0.62; 3.91)
50–75 (vs. Less than 10)	0.088	0.56 (0.28; 1.09)	0.773	1.20 (0.34; 4.26)
75 or More (vs. Less than 10)	--	--	--	--
**Family financial support**, Yes (vs. No)	0.013*	2.84 (1.24; 6.46)	0.007*	6.36 (1.64; 24.67)

*Statistically significant at 5% level (p-value < 0.05)

## Discussion

This study highlights that the attendance to DRS in BIRDEM Hospital, Bangladesh was very high (88%) among children and young people, but it also acknowledges some barriers to attendance. Several studies have explored the impact of DRS attendance among children and young adults in other countries [4, 18]. Lake et al. examined factors influencing DRS attendance among young adults (18–39 years) with T2DM compared to older adults (> 40 years) in Australia. Barriers for the younger group included screening costs and negative emotions and fear related to DM [19]. A study in the USA found that young people (aged 10–26 years) with T1DM were less likely to attend DRS as they got older and had been living with DM for a longer period [20]. Another study conducted in the UK identified barriers to DRS in young adults (< 35 years) with T1DM and T2DM. The main barriers were lack of appointment flexibility, impact of dilating eye drops and anxiety associated with the risk of developing DR [4]. A study in Northern Ireland (NI) by Cushley et al. investigated DRS attendance rates among young people aged 12–26 years and found that 83.2% had good-moderate attendance [21]. The study found that young people under 15 years were more likely to attend DRS than those aged 16–26 years, likely due to greater parental involvement. Individuals with a shorter duration of DM (< 5years) were more likely to attend DRS, while those with longer DM duration showed lower attendance. These results were comparable to our findings in Bangladesh. In NI, those who regularly attended DM clinics were more likely to attend DRS, highlighting the importance of continuity in DM care and education. Lower HbA1c levels were associated with better DRS attendance, emphasising the importance of well-managed DM. In NI, gender and DM type were not predictors for DRS attendance, whereas our study found than females and people who had T2DM were more likely to attend their DRS appointments. Respondents in NI expressed a desire for more educational materials about the importance of regular eye screening, making information more accessible and understandable. Later appointment times, online booking, and appointments during routine DM visits were potential enablers [21].

There’s an overlap in challenges faced by DRS attendance in Bangladesh compared to NI, such as access to services and conflicting schedules or inappropriate appointment times. One notable distinction was that in NI, there were complaints about poor communication, whereas most participants in Bangladesh expressed satisfaction with the service and treatment received. Overall, it’s crucial to consider implementing services that align with people’s schedules, and addressing accessibility to services is important. In Bangladesh, where traveling long distances for appointments is common, patients in NI still perceive this as an issue, despite no specific data on distance to the hospital being captured.

### Strengths

Our study demonstrates a successful DRS programme in Bangladesh, marked by high attendance rates. The inclusion of a pilot study prior to the main data collection underscores the thoroughness in refining the survey. Also, identification of specific barriers, comparison of perceptions, insights into the pandemic’s impact and detailed analyses of demographic and clinical parameters enhances insights into DRS attendance.

### Limitations

We selected BIRDEM as our study site because it is the main centre in Bangladesh providing care for children and young adults with DM. Its high patient volume and capacity to serve individuals from both rural and urban areas made it a suitable choice, with availability also being a contributing factor. However, we acknowledge that including additional sites, particularly in rural areas, would enhance the generalisability of the study findings and should be considered for future studies. By not maintaining a sample size for both groups (attendees versus non-attendees), it reduces the statistical power, introduces potential bias, and affects the interpretation and generalisability of the results. Additionally, our definition of non-attendance also differed from the similar study conducted in NI due to differences in appointment recall procedures. Most participants were recruited from urban cities such as Dhaka, potentially leading to a skewed representation that does not fully cover the diversity of experiences in more rural regions. Therefore, caution should be considered in extending the study’s conclusions to populations in rural settings, and future research activities should aim for a broader and more inclusive representation.

## Conclusions

The attendance rates for DRS among children and young people with DM in Bangladesh are very high. The presence of a one-stop/holistic clinic offers convenience for attendees, the positive hospital experiences and a targeted campaign may have successfully boosted attendance. This study has also identified some challenges to DRS attendance, with long travel distances being a common barrier, particularly for those in rural areas. To address these challenges, enhancing appointment flexibility and implementing remote screening services through telemedicine could be highly effective. Telemedicine has already proven transformative in making DRS more accessible, efficient, and convenient. Integrating telemedicine into schools, managed by school nurses or teachers, could further improve accessibility for children with DM, alleviating scheduling concerns for both parents and students. Future research should explore the feasibility of implementing these services within school settings. 

Moreover, establishing electronic health systems is crucial for efficient monitoring of patients with DM in Bangladesh. Strengthening the education of both patients and healthcare providers about the importance of regular DRS attendance is critical. Effective communication strategies should include clear protocols for reaching out to patients who miss appointments, as well as using culturally appropriate methods to convey the importance of eye health to those with diabetes.

In summary, while our study highlights good attendance rates and positive experiences at the DRS in Dhaka, it also brings attention to barriers that require further exploration. Future research should build on these findings by investigating the experiences of individuals from rural areas, thereby enhancing the generalisability of results across different populations in Bangladesh. This work contributes to the growing body of literature on DRS attendance by identifying unique regional challenges and proposing practical solutions that can be tailored to various settings.

Furthermore, systems using AI to detect DR are evolving and could also play a transformative role, particularly in resource-limited settings. Such technologies can reduce the burden on healthcare systems while improving screening accuracy and accessibility. A recent study in Bangladesh examined the use of AI for detecting DR in a children and young adults [22]. Despite the algorithm being trained on adult data, the Cybersight AI system successfully identified DR and cases requiring referral. Its high specificity is crucial in low-resource environments to prevent over-referrals [22]. AI has the potential to ease the burden on constrained medical resources in managing DM care for children in areas with limited resources, while also improving adherence to attendance.

## Data Availability

All data generated or analysed during this study are included in this published article [and its supplementary information files].

## Declarations

None to declare.

## Abbreviations

BADAS: Diabetic Association of Bangladesh
BIRDEM: Bangladesh Institute of Research and Rehabilitation in Diabetes, Endocrine, and Metabolic Disorders
PDRC: Pediatric Diabetes Care and Research Centre
DM: Diabetes Mellitus
DRS: Diabetic Retinopathy Screening
AI: Artificial Intelligence
T1DM: Type 1 Diabetes Mellitus
T2DM: Type 2 Diabetes Mellitus
DR: Diabetic Retinopathy
LFAC: Life for a Child
CDIC: Changing Diabetes in Children
LMICs: Low- and Middle-Income Countries
SD: Standard Deviation
IQR: Interquartile Range
OR: Odds Ratio
CI: Confidence Intervals
NI: Northern Ireland
